# Paleoindian Unifacial Stone Tool ‘Spurs’: Intended Accessories or Incidental Accidents?

**DOI:** 10.1371/journal.pone.0078419

**Published:** 2013-11-13

**Authors:** Metin I. Eren, Thomas A. Jennings, Ashley M. Smallwood

**Affiliations:** 1 Department of Anthropology, Marlowe Building, University of Kent, Canterbury, United Kingdom; 2 Department of Archaeology, Cleveland Museum of Natural History, Cleveland, Ohio, United States of America; 3 Department of Anthropology, University of West Georgia, Carrollton, Georgia, United States of America; Bristol University, United Kingdom

## Abstract

Paleoindian unifacial stone tools frequently exhibit distinct, sharp projections, known as “spurs”. During the last two decades, a theoretically and empirically informed interpretation–based on individual artifact analysis, use-wear, tool-production techniques, and studies of resharpening–suggested that spurs were sometimes created intentionally via retouch, and other times created incidentally via resharpening or knapping accidents. However, more recently Weedman strongly criticized the inference that Paleoindian spurs were ever intentionally produced or served a functional purpose, and asserted that ethnographic research “demonstrates that the presence of so called ‘graver’ spurs does not have a functional significance.” While ethnographic data cannot serve as a direct test of the archaeological record, we used Weedman’s ethnographic observations to create two quantitative predictions of the Paleoindian archaeological record in order to directly examine the hypothesis that Paleoindian spurs were predominantly accidents occurring incidentally via resharpening and reshaping. The first prediction is that the frequency of spurs should increase as tool reduction proceeds. The second prediction is that the frequency of spurs should increase as tool breakage increases. An examination of 563 unbroken tools and 629 tool fragments from the Clovis archaeological record of the North American Lower Great Lakes region showed that neither prediction was consistent with the notion that spurs were predominately accidents. Instead, our results support the prevailing viewpoint that spurs were sometimes created intentionally via retouch, and other times, created incidentally via resharpening or knapping accidents. Behaviorally, this result is consistent with the notion that unifacial stone tools were multifunctional implements that enhanced the mobile lifestyle of Pleistocene hunter-gatherers.

## Introduction

There is little doubt that throughout most the 20th century research on North American Paleoindian flaked stone tools has traditionally centered on bifaces, especially fluted projectile points ([1]; [2]∶142). There were, and still are, very good reasons for this focus. Questions involving mobility, raw material selection, core reduction, settlement patterns, and gearing-up can be profitably examined (e.g., [3]; [4]; [5]; [6]; [7]; [8]; [9]). Fluted point and biface flaking, resharpening, and shape patterns can reveal stylistic signatures, evolutionary trends, adaptation to environments, and evidence of cultural transmission (e.g., [10]; [11]; [12]; [13]; [14]; [15]). Furthermore, the study of bifaces and fluted points is one avenue for understanding aspects of Paleoindian tool-making and tool-using efficiency and function (e.g., [16]; [17]; [18]; [19]; [20]; [21]; [22]; [23]; [24]; [25]; [26]; [27]; [28]) and, perhaps, even less utilitarian aspects of Paleoindian life (e.g., [29]; [30]).

In more recent years, however, Paleoindian archaeologists have come to realize that while the study of bifacial implements is vital for answering numerous questions, constructing more holistic models of Paleoindian behavior requires the consideration of other stone tool classes ([1]; [2]). Thus, while there were early exceptions to the priority given to the study of projectile points (e.g., [31]; [32]; [33]), over the last 30 years there has been an increase of research focusing more heavily on other Paleoindian stone tool classes, especially unifacially flaked stone tools (e.g., [2]; [34]; [35]; [36]; [37]; [38]; [39]; [40]; [41]; [42]; [43]; [44]; [45]; [46]; [47]; [48]; [49]; [50]; [51]; [52]; [53]; [54]; [55]; [56]; [57]; [58]).

The tools of mobile foragers often possess properties that were dictated by, as well as helped enhance, a mobile lifestyle. Mobile tools offered the properties of portability, durability, maintainability, reliability, efficiency (effectiveness), and multi-functionality (versatility and flexibility) ([2]; [59]; [60]; [61]; [62]; [63]; [64]; [65]; [66]; [67]; [68]; [69]; [70]; [71]). Since unifacial stone tools were important, and often major, components of the Paleoindian mobile toolkit ([41]; [46]), and since Paleoindians were highly mobile ([72]; [73]), archaeologists have inferred from the archaeological record that unifacial tools were often designed for portability ([3]) and maintainability ([41]). However, one aspect Paleoindian unifacial tool technology remains unresolved. With regard to the property of multi-functionality of these tools, one longstanding question has been chronically debated. Were distinct, sharp projections–known as “spurs”–on Paleoindian unifacial stone tools functional “accessories” intentionally shaped by prehistoric flintknappers for the purpose of facilitating tasks such as “tattooing, piercing, ripping or tearing hide, and engraving bone, antler, wood, and ivory” ([74]∶731), or were spurs non-functional phenomena, the incidental formation of which is best explained by tool resharpening or knapping accidents?

The traditional answer to this question, assumed by numerous Paleoindian lithic analysts throughout much of the 20th century, was that spurs were intentionally created functional accessories. To many of these early researchers this conclusion was so intuitively obvious that the “spurred [*insert unifacial tool type here*]” was a commonly listed tool category (e.g., [75]; [76]; [77]). However, during the last two decades, a more theoretically and empirically informed interpretation–based on individual artifact analysis, use-wear, tool-production techniques, and studies of resharpening–suggested that spurs were sometimes created intentionally via retouch, and other times created incidentally via resharpening or knapping accidents ([35]; [36]; [39]; [45]; [52]; [54]; [55]; [56]; [78]).

Weedman ([74]∶731) strongly criticized the inference that Paleoindian spurs were *ever* intentionally produced or served a functional purpose, asserting that ethnographic research “demonstrates that the presence of so called ‘graver’ spurs does not have a functional significance.” Her stance is based on two sets of ethnographic observations. First, the ethnoarchaeological research of Clark and Kurashina ([79]) and Nissen and Dittemore ([80]) suggested to her that spurs could form incidentally via resharpening unifacial stone tools. Indeed, Weedman’s own ethnographic data from four distinct villages in Ethiopia–whose people did not intentionally create this functional accessory ([74]∶739)–show that the percentage of used-up scrapers exhibiting a spur ranged from 2.9% to 19.0%. While these observations underscore the possibility that some Paleoindian spurs were created incidentally via resharpening, they do not falsify the notion that prehistoric spurs could also have been created intentionally for functional purposes. Nevertheless, Weedman ([74]∶732) advocated that “spurs are the result of reshaping or resharpening.” Nearly 20 years earlier, Grimes et al. ([81]∶165) asserted as much:

“Spurred scrapers seem to represent one extreme of a spectrum of lateral edge modifications which includes constriction, notching, and ventral thinning. These modifications probably represent alternative means of adapting end scrapers to a haft. Spurred end scrapers may be the end product of periodic resharpening and bit attrition of notched specimens. In effect, this process would continually reduce the bit to notch distance, to the point where they ultimately converged, producing the characteristic spur. If this hypothesis is correct, spurred end scrapers may be a ‘diagnostic’ Palaeo-Indian artifact only in the sense that they are the by-product of a strategy for maximizing to longevity, a trait associated with Palaeoindian lithic technology.”

The second set of observations upon which Weedman ([74]) based her stance involved not only the act of resharpening, but the practical experience of the ethnographic knappers who were doing the resharpening. Her ethnographic data showed that “villages with the highest percentage of spurred scrapers have more hideworkers with three or fewer years or more than 30 years of experience knapping” ([74]∶738). Thus, the ethnographic record suggests there exists both a mechanism (i.e., resharpening) for the incidental creation of spurs, as well as a variable (i.e., age/experience) that explains how that mechanism may vary to create the low to high proportions of spurs in different assemblages. Based on this, Weedman concluded “ethnoarchaeological evidence eliminates a functional explanation for spurs” ([74]∶741–742), and hence, “it is quite probable that the presence of spurs on scrapers in archaeological assemblages worldwide contexts represent not formally made tools, but accidents” ([74]∶741).

Distinguishing between accidental and intentional spurs has important implications for understanding Paleoindian technological organization. If Weedman ([74]) is correct and spurs on Paleoindian unifaces are accidents of resharpening, then spur presence is related only to reduction intensity. Spurs can be used as a direct indicator of curation but are themselves not related to other aspects of technological organization. Alternatively, if spurs are intentionally produced for tool use, then spur presence can be used to understand other facets of Paleoindian technological organization including the role of tool multifunctionality.

While ethnographic analogy can provide a range of *possible* explanations for particular tool morphologies, documenting and understanding a pattern in ethnographic data is not a direct test of archaeological data ([82]; [83]). With regard to the overall incidental or intentional nature of Paleoindian spurs, it cannot be assumed that because ethnographic spurs from one small region in Africa are primarily created incidentally, Paleoindian spurs, or those present in “worldwide contexts,” were therefore also created incidentally. Any conclusion made about Paleoindian behavior can only come from a test of the Paleoindian archaeological record itself. So, while we disagree that Weedman’s ([74]) conclusions can be universally applied, her ethnographic observations are still scientifically testable. By using those ethnographic observations as a foundation for the creation of quantitative predictions of the Paleoindian archaeological record, this is the first paper to directly test the hypothesis that Paleoindian spurs were accidents occurring incidentally via resharpening and reshaping.

We identify two predictions stemming from Weedman’s ([74]) hypothesis. The first prediction is that if Paleoindian spurs were predominantly the incidental result of resharpening as Weedman ([74]) suggests, then we can predict that there will be increasingly higher frequencies of spurs present in sets of unifacial stone tools that are relatively more resharpened than there will be in sets of unifacial stone tools that are relatively less resharpened. This prediction is based on two reasons. First, as Grimes et al. ([81]) notes above, spurs may be the result of resharpening and bit attrition of notched specimens. In effect, the process would continually have reduced the working edge to notch distance, to the point where they ultimately converged, producing a spur. Second, as a unifacial stone tool is resharpened and becomes progressively smaller, rounder, and thicker, more force is required for retouch. The combination of increased retouch force and increasingly undesirable tool shape fosters a situation where mistakes (like spurs) are not only more likely to happen, but one in which those mistakes are harder to rectify (see [46]∶328). In aggregate, these two reasons allow us to reasonably predict, on the population level, that tools resharpened to a greater degree would be more likely to possess spurs when discarded than those tools that are relatively less resharpened.

Second, because “spurs frequently occur in assemblages with high breakage rates” ([74]∶741), if spurs are primarily the result of resharpening accidents we can predict to see a significant positive relationship between the percentage of broken unifacial stone tools at a site and spur frequency. If we do not see these patterns in the Paleoindian archaeological record, then the notion that Paleoindian spurs were primarily the accidental result of resharpening is not supported, and instead, they might be the result of intentional shaping.

## Materials and Methods

### Samples

To examine the first prediction data were recorded from 563 unbroken unifacial stone tools from seven Clovis sites in the North American Lower Great Lakes region. The analyzed class of “unifacial stone tools” was explicitly defined following Eren et al. ([43]). To examine the second prediction an additional 629 specimens were counted (**TABLE 1**).

**Table 1:** Number of unifacial stone tool specimens (total, unbroken, and broken) recorded for this study relative to the actual or estimated number of specimens per assemblage.

Site	Number of recordedunifacial stone toolspecimens	Number of recordedunbroken unifacialstone tool specimens	Number or recodedbroken unifacialstone tool specimens	Actual (A) or Estimated(E) number of specimensin the assemblage
Arc	250	135	115	700 (E)
Butler	70	63	7	100 (E)
Gainey	64	31	33	90 (E)
Leavitt	71	33	38	71 (A)
Paleo Crossing	401	160	241	401 (A)
Potts	123	41	82	123 (E)
Udora	210	97	113	Unknown

These specimens are curated at the Cleveland Museum of Natural History, Cleveland, Ohio (Paleo Crossing site); the State Museum of New York, Albany, New York (Potts site); the Royal Ontario Museum, Toronto, Canada (Udora site); the Museum of Anthropology at the University of Michigan, Ann Arbor, Michigan (Leavitt site); and by the private collectors Donald B. Simons in Michigan (Butler, Gainey sites) and Stanley Vanderlaan in New York (Arc site). No permits were required for the described study, which complied with all relevant regulations. All that was needed was permission from the museums or private collectors where the archaeological specimens are curated, and this was given freely.

The regional designation of these sites as “Clovis” is based on the presence of diagnostic artifacts of the Clovis period, namely fluted projectile points with relatively parallel lateral edges and the lack of full-face flutes. Furthermore, the Paleo Crossing site possesses a significant prismatic blade component ([84]), and the Arc site exhibits overshot flakes ([22]), both characteristics of Clovis sites in southern regions of North America. Two of the sites, Paleo Crossing and Sheriden Cave, have yielded average radiocarbon ages of 10,980±75 B.P. and 10,915±30 B.P., respectively ([85]; [86]).

Shott ([54]) suggested that the Leavitt site possessed “Parkhill style” projectile points, a Clovis point variant assumed to occur slightly later in time (though there is no chronometric evidence supporting this assertion in the Lower Great Lakes). Having investigated the Leavitt fluted projectile points ourselves, we agree with others (e.g. [87]; [88]) who classify the points, thus the site, as Clovis. Indeed, even Shott ([54]∶102) suggested that site exhibited similarity to Clovis with respect to particular variables. However, given that Shott ([54]∶102) ultimately concluded that the site is the “earliest Parkhill phase assemblage under study”, and given that the Parkhill Paleoindian phase is estimated to have commenced ca. 10,700 B.P. ([89]), even if one wishes to classify Leavitt as a Parkhill phase site rather than a Clovis one, a case can be made that the site is still representative of the initial colonization stages of the region.

### A Quantitative Definition and Measurement of “Spurs”

**Figure 1:**
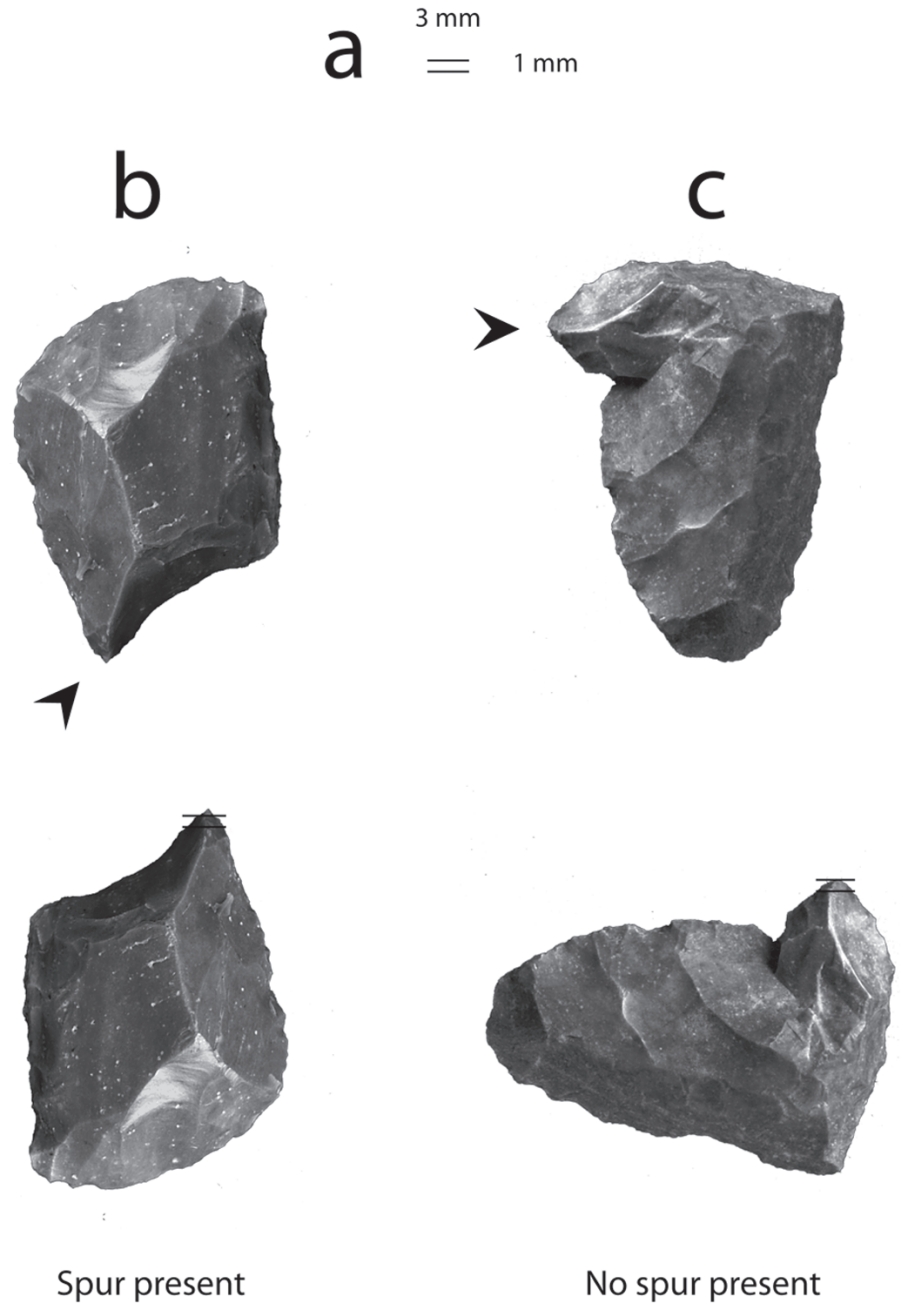
Two examples of how the presence of spurs was determined quantitatively. A box of 3(a). A spur was any projection no wider than 3 mm, but at least 1 mm long (b) (compare with c).

A spur was defined as any projection no wider than 3 mm, but at least 1 mm long. A box of these dimensions was rendered on paper for an easy, objective, replicable means of quickly assessing whether projections met our metric criteria of a spur (see **figure 1**).

### Tool Resharpening Proxies

In order to assess whether spurs increased in frequency as resharpening advanced, two unifacial stone tool resharpening proxies were used. First, following Buchanan and Collard ([17]; [90]) and Iovita ([91]; [92]), we used tool size as a proxy for tool resharpening extent (mass loss). Although tool size is not always an appropriate proxy for stone tool reduction, for flaked tools that were likely hafted–like Clovis unifaces ([40]; [52]; [54]; [55])–it seems reasonable to use size as a rough proxy for reduction on the grounds that smaller tools are more likely to have been resharpened than larger tools within the same tool class ([90]∶262; see also [93]). Size itself can be measured numerous ways, and here simple tool mass (g) was used.

**Figure 5:**
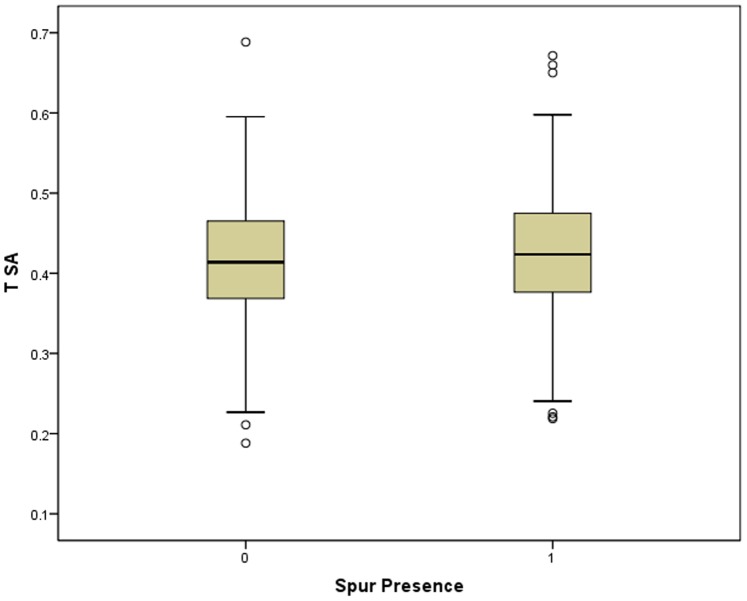
Box plots of TSA vs. spur presence/absence.

The second proxy used for tool resharpening was tool shape. A number of lithic analysts have shown that the allometry of unifacial stone tools changes as resharpening advances (e.g., [94]; [95]); namely, tool thickness plays a progressively larger role in tool shape because tool thickness is minimally effected by retouch ([2]; [5]; [50]). Indeed, Eren ([41]) has already shown for the data used here that there is a statistically significant relationship between tool size and shape and, specifically, that as tools got smaller, they also got rounder. Like tool size, tool shape (roundness) can be measured numerous ways, and here shape was assessed as geometric mean size-adjusted thickness (Tsa) ([41]). Size-adjustment of the data proceeds on a specimen-by-specimen basis, dividing each variable in turn by the geometric mean of all variables for that individual specimen. This procedure effectively equalizes the volume of all specimens in a sample, creating a dimensionless scale-free variable while maintaining the original shape information of the data ([96]∶854). The variables measured on each specimen to calculate geometric mean size-adjusted thickness were length, width and thickness (see [41]:Figure 5). Length was measured as the distance parallel to the axis of percussion between the platform (or most proximal point) and the most distal point on the specimen. Width was measured as the distance between the two lateral edges of the specimen at the mid-point of and perpendicular to the length measurement. Thickness was measured as the distance between the dorsal and ventral faces of the specimen at the same location as the width measurement.

### Unifacial Stone Tool Breakage Rates Per Site

In order to estimate the number of broken unifacial stone tools as a percentage of all unifacial stone tools at an archaeological site, the number of broken specimens was simply divided by the number of all specimens. Although the samples of broken vs. unbroken specimens were acquired from a larger population of unknown size (because none of the sites have been fully excavated), the robust sample sizes that were counted suggest that we were at least approaching an accurate estimate of the true ratio of broken to unbroken tools (**TABLE 1**).

### Statistical Analyses

To thoroughly compare the relationships between tool size, shape, and breakage and the presence of spurs, we employ multiple, independent statistical tests. Shapiro-Wilk tests demonstrate that tool mass is not normally distributed (*W* = 0.762, df = 560, p<0.001) and size-adjusted-thickness is normally distributed (*W* = 0.996, df = 560, p = 0.247). Therefore, we chose to use different statistical tests to assess comparisons involving these two measures. To test for differences in group means in comparisons involving mass, we use the Mann-Whitney U and Kruskal-Wallis non-parametric tests, and for comparisons involving size-adjusted-thickness, we use T-test and Analysis of Variance (ANOVA) with subsequent pair-wise Bonferroni comparisons (for significant results only). To test for correlations between these variables and spur count and spur presence, we performed Spearman’s rho correlation analyses.

## Results

### Does Spur Frequency Increase as Resharpening Advances?

**Figure 2:**
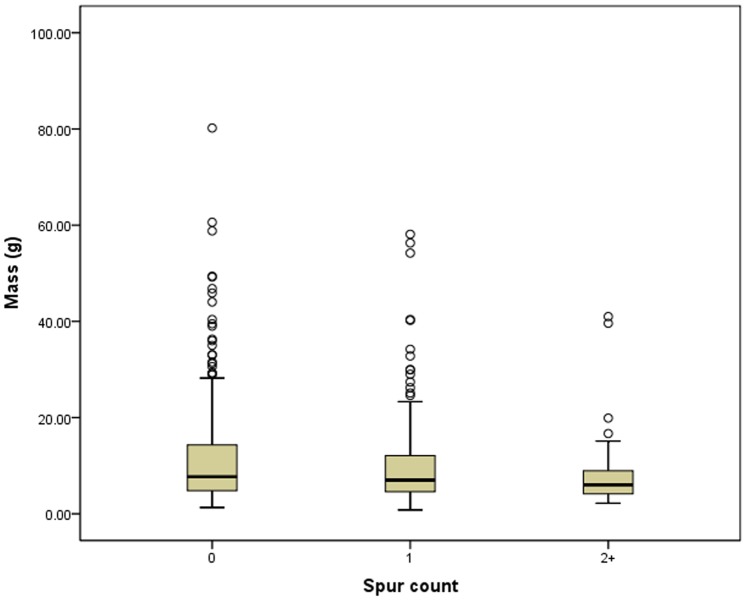
Box plots of tool mass vs. spur count.

The relationship between tool mass (as a proxy for reduction) and tool spurs was evaluated using both total spur count and spur presence/absence. Beginning with spur count, tool mass does significantly (Kruskal Wallis chi^2^ = 6.137; df = 2; p = 0.046) decrease with increasing numbers of spurs (**TABLE 2**, **FIGURE 2**). This result **does** support the hypothesis that most spurs are created incidentally or accidentally via resharpening. There also appears to be a significant (p = 0.018), negative correlation between tool mass and spur count, however, the Spearman’s rho of −0.100 shows this correlation to be extremely weak. This result **is equivocal** with regard to the notion that most spurs are created incidentally or accidentally via resharpening. To further test this relationship, a series of Spearman’s rho correlation analyses were applied to ranked groupings of the tool dataset. The 563 unifacial stone tools were divided into 10 groups of 56 specimens each (except for the last group, which has 59 specimens). Because some unifacial tools have more than one spur, and others have none, the total number of spurs can be counted and divided by the number of unifacial tools in each group to get a “spurs per uniface” value (**TABLE 3**). As unifacial stone tool size gets smaller (reduction proceeds) the spurs-per-uniface value significantly increases (ρ = 0.644, p = 0.044). This result **does** support the notion that spurs are created incidentally or accidentally via resharpening. The problem with groups of equal sample size is that the range of masses becomes smaller in each group. Regrouping the tools into groups of equal, 5 g mass ranges gives 6 groups (**TABLE 4**). Spearman’s rho correlation analysis comparing these 6 groups to spurs-per-uniface yields a significant relationship that **does** support the notion that a greater number of spurs are a result of resharpening (ρ = 0.943, p = 0.005).

**Table 2:** Comparison of tool mass and spur count.

n	Mass Avg. (g)	Standard Deviation	Spur Count
321	11.5	10.6	0
192	10	9.3	1
47	8.5	7.9	2+
	Kruskal-Wallis chi^2^ = 6.137; df = 2; p-value = 0.046		
	Spearman’s ρ = −0.100, p = 0.018		

**Table 3:** As unifacial stone tool size gets smaller (reduction proceeds) the spurs-per-uniface value significantly increases (ρ = 0.644, p = 0.044).

SizeGroups	#Spurs	MassRange	n	Spurs/Uniface
1 (Largestspecimens)	20	80.2–22.9	56	0.3571
2	22	22.72–14.9	56	0.3929
3	26	14.8–11.6	56	0.4643
4	24	11.5–8.8	56	0.4286
5	37	8.8–7.2	56	0.6607
6	36	7.2–6.1	56	0.6429
7	24	6.1–5	56	0.4286
8	38	5–4.4	56	0.6786
9	32	4.3–3.4	56	0.5714
10 (Smallestspecimens)	34	3.4–0.8	59	0.5763

This result supports the notion that spurs were created incidentally or accidentally via resharpening. In this instance, the size groups were of equal sample size.

**Table 4:** As unifacial stone tool size gets smaller (reduction proceeds) the spurs-per-uniface value significantly increases (ρ = 0.943, p = 0.005).

SizeGroups	#Spurs	MassRange	n	Spurs/Uniface
1 (Largestspecimens)	17	>25 g	46	0.3696
2	7	20 g–25 g	23	0.3043
3	16	15 g–20 g	41	0.3902
4	42	10 g–15 g	86	0.4884
5	111	5 g–10 g	203	0.5468
6 (Smallestspecimens)	100	<5 g	164	0.6098

This result supports the notion that spurs were created incidentally or accidentally via resharpening. In this instance, the mass range of each group was equal.

**Figure 3:**
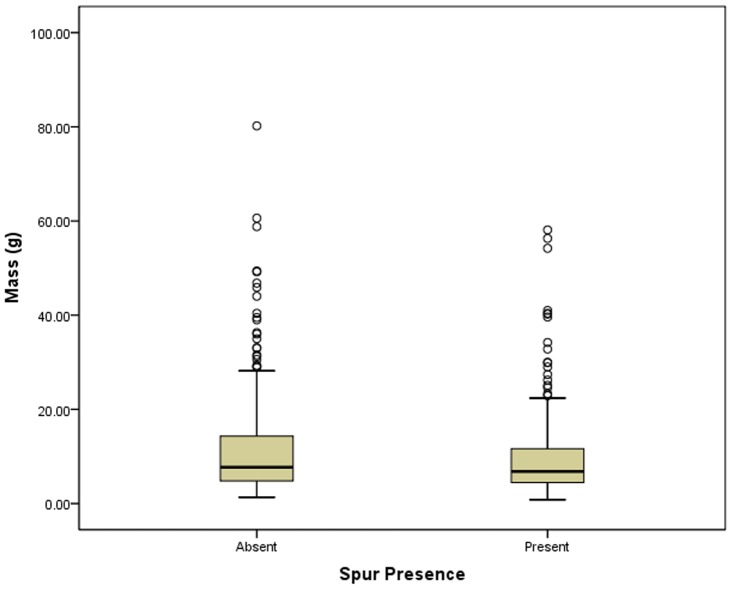
Box plots of tool mass vs. spur presence/absence.

Turning from spur count to spur presence/absence, there a significant (Mann-Whitney U = 34,310.000; p = 0.032) relationship between tool mass and spur presence/absence (**TABLE 5**, **FIGURE 3**). This significance is driven by differences in the heavy range of scrapers, and it **does** support the notion that spurs are created incidentally or accidentally via resharpening. There are more very large scrapers (with masses larger than 30 g) that have no spurs. However, at the opposite end, it is clear that many small scrapers also have no spur. There does appear to be a significant (p = 0.032), negative correlation between tool mass and spur presence, however, the Spearman’s rho of −0.090 shows this correlation to be extremely weak. This result **is equivocal** with regard to the notion that most spurs are created incidentally or accidentally via resharpening. Spearman’s rho correlation analyses of the 10 equal-count groups (**TABLE 6**) shows that as unifacial stone tool size gets smaller the percentage of unifacial stone tools possessing a spur increases, but the relationship is not significant (ρ = 0.542, p = 0.106). This result **does not** support the notion that most spurs are created incidentally or accidentally via resharpening. Spearman’s rho analyses of equal-mass-range groups (**TABLE 7**) shows that as mass decreases, the percentage of unifacial stone tools possessing a spur increases, and the relationship is significant (ρ = 0.829, p = 0.042). This result **does** support the notion that most spurs are created incidentally or accidentally via resharpening.

**Table 5:** Comparison of tool mass and spur presence/absence.

n	Mass Avg. (g)	Standard Deviation	Spur Presence
321	11.5	10.6	Absent
239	9.7	9	Present
	Mann-Whitney U = 34,310.000; p-value = 0.032		
	Spearman’s ρ = −0.090, p = 0.032		

**Table 6:** As unifacial stone tool size gets smaller (reduction proceeds) the % of unifaces with a spur does not significantly increase (ρ = 0.542, p = 0.106).

Size Groups	# Unifaces With a Spur	Mass Range	n	% of Unifaces With Spur
1 (Largest specimens)	18	80.2–22.9	56	32%
2	18	22.72–14.9	56	32%
3	24	14.8–11.6	56	42%
4	21	11.5–8.8	56	37%
5	30	8.8–7.2	56	53%
6	28	7.2–6.1	56	50%
7	18	6.1–5	56	32%
8	30	5–4.4	56	53%
9	25	4.3–3.4	56	44%
10 (Smallest specimens)	27	3.4–0.8	59	45%

This result does not support the notion that spurs were created incidentally or accidentally via resharpening. In this instance, the size groups were of equal sample size.

**Table 7:** As unifacial stone tool size gets smaller (reduction proceeds) the the % of unifaces with a spur significantly increases (ρ = 0.829, p = 0.042).

Size Groups	# Unifaces With a Spur	Mass Range	n	% of Unifaces With Spur
1 (Largest specimens)	15	>25 g	46	32%
2	7	20 g–25 g	23	30%
3	13	15 g–20 g	41	31%
4	37	10 g–15 g	86	43%
5	89	5 g–10 g	203	43%
6 (Smallest specimens)	79	<5 g	164	48%

This result supports the notion that spurs were created incidentally or accidentally via resharpening. In this instance, the mass range of each group was equal.

**Figure 4:**
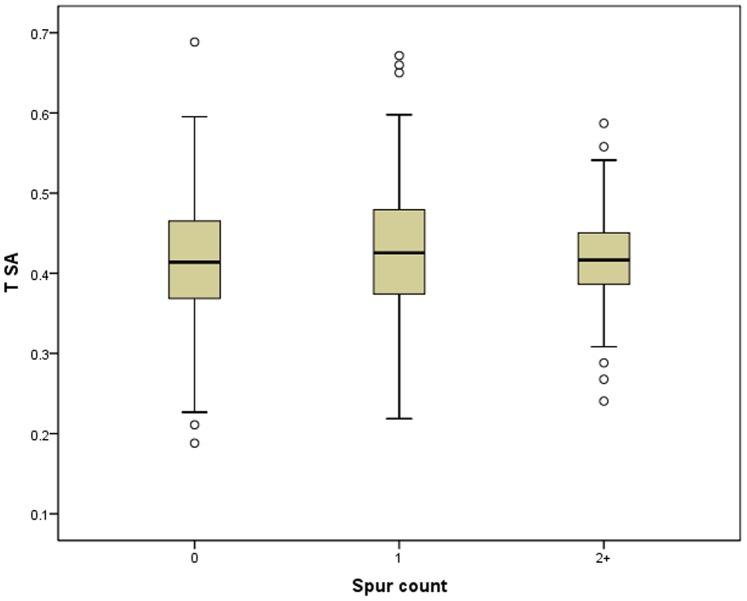
Box plots of TSA vs. spur count.

The relationship between tool size-adjusted-thickness (as a second proxy for reduction, Tsa) and tool spurs was evaluated using both total spur count and spur presence/absence. Beginning with spur count, Tsa does not significantly vary (ANOVA p = 0.245) with spur count (**TABLE 8**, **FIGURE 4**). This result **does not** support the notion that most spurs are created incidentally or accidentally via resharpening. Likewise, there is no significant (Spearman’s ρ = 0.053, p = 0.215) correlation between Tsa and spur count. This result **does not** support the notion that most spurs are created incidentally or accidentally via resharpening. Spearman’s rho correlation analyses of equal-count groups (**TABLE 9**) shows no significant relationship between Tsa and spurs-per-uniface (ρ = 0.394, p = 0.260). This result **does not** support the notion that most spurs are created incidentally or accidentally via resharpening. Spearman’s rho correlation analyses of equal-mass-range groups (**TABLE 10**) shows no significant relationship between Tsa and spurs-per-uniface (ρ = .257, p = 0.623). This result **does not** support the notion that most spurs are created incidentally or accidentally via resharpening.

**Table 8:** Comparison of TSA and spur count.

n	TSA Mean	Standard Deviation	Spur Sount
321	0.415	0.74	0
192	0.426	0.80	1
47	0.416	0.76	2+
	ANOVA p-value = 0.245		
	Spearman’s ρ = 0.053, p = 0.215		

**Table 9:** As unifacial stone tools get thicker and rounder (size-adjusted thickness, Tsa, increases) the spurs-per-uniface value does not significantly increase (ρ = 0.394, p = 0.260).

Tsa Groups	# Spurs	Tsa Range	n	Spurs/Uniface
1 (Rounderspecimens)	32	.4739–.2649	56	0.5714
2	33	.2642–.2287	56	0.5892
3	25	.2286–.2077	56	0.4464
4	36	.2076–.1876	56	0.6428
5	29	.1873–.1759	56	0.5178
6	37	.1756–.1618	56	0.6607
7	21	.1615–.1456	56	0.3750
8	24	.1454–.1292	56	0.4285
9	30	.1292–.1055	56	0.5357
10 (Flatterspecimens)	27	.1052–.0353	59	0.4576

This result does not support the notion that spurs were created incidentally or accidentally via resharpening. In this instance, the size groups were of equal sample size.

**Table 10:** As unifacial stone tools get thicker and rounder (size-adjusted thickness, Tsa, increases) the spurs-per-uniface value does not significantly increase (ρ = .257, p = 0.623).

Tsa Groups	# Spurs	Tsa Range	n	Spurs/Uniface
1 (Rounderspecimens)	15	>0.30	23	0.6521
2	30	.30–.25	54	0.5555
3	54	.25–.20	114	0.4736
4	105	.20–.15	186	0.5645
5	62	.15–.10	137	0.4525
6 (Flatterspecimens)	26	.10–.05	46	0.5652

This result does not support the notion that spurs were created incidentally or accidentally via resharpening. In this instance, the Tsa range of each group was equal.

Moving from spur count to spur presence/absence, there is no significant relationship (T-test p = 0.142) between average Tsa and spur presence/absence (**TABLE 11**, **FIGURE 5**). This result **does not** support the notion that most spurs are created incidentally or accidentally via resharpening. Likewise, there is no significant correlation (Spearman’s ρ = 0.061, p = 0.152) between Tsa and spur presence/absence. This result **does not** support the notion that most spurs are created incidentally or accidentally via resharpening. Spearman’s rho correlation analyses of equal-count groups (**TABLE 12**) shows no significant relationship between Tsa and the percentage of unifacial stone tools possessing a spur (ρ = 0.470, p = 0.171). This result **does not** support the notion that most spurs are created incidentally or accidentally via resharpening. Spearman rho correlation analyses of equal-mass-range groups (**TABLE 13**) shows no significant relationship between Tsa and spurs-per-uniface (ρ = 0.600, p = 0.208). This result **does not** support the notion that most spurs are created incidentally or accidentally via resharpening.

**Table 11:** Comparison of TSA and spur presence.

n	TSA Mean	Standard Deviation	Spur Presence
321	0.415	0.74	Absent
239	0.424	0.78	Present
	T-test p-value = 0.142		
	Spearman’s ρ = 0.061, p = 0.152		

**Table 12:** As unifacial stone tools get thicker and rounder (size-adjusted thickness, Tsa, increases) the % of unifaces with a spur does not significantly increase (ρ = 0.470, p = 0.171).

Tsa Groups	# Unifaces With a Spur	Tsa Range	n	% of Unifaces With Spur
1 (Rounder specimens)	25	.4739–.2649	56	0.4464
2	31	.2642–.2287	56	0.5535
3	21	.2286–.2077	56	0.3750
4	29	.2076–.1876	56	0.5178
5	22	.1873–.1759	56	0.3928
6	25	.1756–.1618	56	0.4464
7	18	.1615–.1456	56	0.3214
8	21	.1454–.1292	56	0.3750
9	26	.1292–.1055	56	0.4642
10 (Flatter specimens)	20	.1052–.0353	56	0.3571

This result does not support the notion that spurs were created incidentally or accidentally via resharpening. In this instance, the size groups were of equal sample size.

**Table 13:** As unifacial stone tools get thicker and rounder (size-adjusted thickness, Tsa, increases) the % of unifaces with a spur does not significantly increase (ρ = 0.600, p = 0.208).

Tsa Groups	# Unifaces With a Spur	Tsa Range	n	% of Unifaces With Spur
1 (Rounder specimens)	13	>0.30	23	0.5652
2	26	.30–.25	54	0.4814
3	46	.25–.20	114	0.4035
4	78	.20–.15	186	0.4193
5	55	.15–.10	137	0.4014
6 (Flatter specimens)	20	.10–.05	46	0.4347

This result does not support the notion that spurs were created incidentally or accidentally via resharpening. In this instance, the Tsa range of each group was equal.

### Does Spur Frequency Increase with Increased Tool Breakage?

Spur frequency does not increase with increased tool breakage (**TABLE 14**). When the breakage percentage of unifacial tools per site is plotted against the percentage of unifacial stone tools with spurs per site there is no relationship (Spearman’s rho = 0.143, p = 0.760). Similarly, when the breakage percentage of unifacial tools per site is plotted against the spurs per uniface value per site there is no relationship (Spearman’s rho = 0.0336, p = 0.939). With regard to our second prediction, the analyzed data **do not support** the hypothesis that spurs were created accidentally during resharpening.

**Table 14:** There is no significant correlation between % of broken unifacial stone tools and spurs-per-uniface value (ρ = 0.0336, p = 0.939) nor % of unifaces with a spur (ρ = 0.143, p = 0.760).

Site	% of broken unifacial stone tools	Spurs/Uniface	% of Unifaces With Spur
Arc	45.6%	0.392	0.344
Butler	10.0%	0.257	0.257
Gainey	51.5%	0.281	0.265
Leavitt	53.5%	0.450	0.380
Paleo Crossing	59.6%	0.381	0.331
Potts	66.6%	0.276	0.235
Udora	53.8%	0.390	0.314

## Discussion

Examination of both the empirical predictions shows that they are inconsistent with the notion that sharp projections on unifacial stone tools were *predominately* created via incidental or accidental mechanisms. The first prediction, spur frequency increases with reduction, can be confidently rejected for this Paleoindian dataset. Using mass as a proxy for reduction produced equivocal results. Some statistical tests suggest that spur count and spur presence/absence significantly vary inversely with mass, but other tests suggest the opposite. When size-adjusted-thickness is used as a proxy for reduction, however, every statistical test yields results which allow us to reject the null hypothesis that spur count or spur presence increase with reduction. Likewise, the second prediction, spur frequency increases with tool breakage, shows that there is no relationship between these two variables.

Why do some of the mass comparisons appear to contradict other results, including all of the size-adjusted-thickness results? In our opinion, the most likely explanation involves the use of mass as a reduction measure. It is highly possible that mass is an imperfect measure of reduction given that initial flake-blank size (mass) likely varied. For example, a scraper made on a small initial flake-blank, but given intentional spurs, would *appear* to be resharpened if only mass is used as a reduction proxy. Size-adjusted-thickness, on the other hand, measures changes in tool shape, possibly providing a more reliable measure of tool reduction intensity that is independent of initial blank size.

Overall, these results do not support Weedman’s assertion that “it is quite probable that the presence of spurs on scrapers in archaeological assemblages worldwide contexts represent not formally made tools, but accidents” ([74]∶741). Instead, our examination of spurs present on unifacial tools made by Clovis foragers in the North American Lower Great Lakes region is consistent with the viewpoint that spurs were at least on occasion created intentionally via retouch. Given that sharp projections of spur-like morphology can arise from either intentional, as demonstrated in this paper, or incidental knapping, as suggested by Weedman ([74]), the status and function of worldwide collections of unifacial stone tool spurs must be empirically established on a case by case basis rather than assumed ([55]∶60). Our results demonstrate that spurs in this Paleoindian sample cannot be explained as *predominately* incidental.

The obvious and perhaps most direct way spur status and function can be demonstrated is via microwear analysis. In spite of several microwear studies that showed spurs to be highly worn with parallel striations indicating the slotting of bone ([75]; [77]∶90; [97]), Weedman ([74]∶723) argued that “there is notably an absence of microwear studies to verify suggested functions for spurs” because “the presence of wear, polish, and striations on spurs may be a consequence of contact with mastic or the hafting itself rubbing on the spur.” As such, the creation and analysis of experimental middle-range data sets might provide tremendous insight as to whether spur microwear resulting from direct versus incidental use is truly equifinal (e.g., [57]). Even then, however, demonstrating specific instances of Paleoindian spur function will probably remain challenging. Loebel’s ([46]) microwear analysis of Clovis endscrapers from Hawk’s Nest (Illinois), Gainey (Michigan), Nobles Pond (Ohio), and Shawnee-Minisink (Pennsylvania) suggested that wear on spurs could form via failed “last-ditch” resharpening attempts as edge angles became too steep to execute satisfactory edge rejuvenation. Thus, microwear resulting from inferred spur functions such as tattooing, piercing, ripping, or tearing hide, and engraving bone, antler, wood, and ivory might be obscured or eliminated entirely. Yet, on a unifacial tool from the Paleo Crossing site (Ohio), Miller ([98]) documented bright, pitted polish confined to lateral margins of flaked spurs indicating it was used to engrave bone or antler. Finally, Waters et al. ([58], see also [99]) report documented use-wear traces on end scrapers from the Gault site (Texas). Based on polish locations and striation directions, Wiederhold [99] argues that some end scrapers served multiple use-functions during their use-lives. One of those important functions produces extensive wear on spurs of 9 out of the 10 end scrapers examined. They do not, however, discuss what tasks these spurs may have been used for.

To our knowledge, the predictions and results presented above constitute the first explicit, quantitative study specifically examining the interpretation of spur production in any prehistoric context. Furthermore, our study contributes to the growing awareness among archaeologists that claims for prehistoric intentionality with regard to lithic technology–positive or negative–must be empirically and quantitatively supported, rather than assumed ([23]; [100]). Ethnographic studies such as those conducted by Weedman ([74]) are important resources for developing the empirical and quantitative hypotheses and predictions necessary for explaining patterns in the archaeological record. In this paper, we investigated Weedman’s conclusions based on ethnographic analysis and identified testable implications for the hypothesis that Paleoindian scraper spurs are an incidental result of tool resharpening. We showed that incidental resharpening cannot entirely explain the presence of spurs on early Paleoindian unifacial tools in the Great Lakes region. Spurs were more likely produced intentionally as part of the multi-use functions of these Clovis unifacial tools. We are not suggesting that all Paleoindian spurs are intentional or even that all Clovis spurs are intentional. In every case, hypotheses of intentionality must be tested. Indeed, given the significant regional variation in Clovis adaptations and technologies across the continent ([7]; [8]; [12]; [72]; [73]; [101]; [102]) it is highly possible that spurs on unifacial Clovis tools were produced for different functions and in different frequencies in the various environments inhabited by Clovis hunter-gatherers. There is likely to be temporal variation in the frequency of spurs as well, and thus the issue of intentional or incidental spur creation should also be examined in Early Archaic or Late Prehistoric contexts of North America.

However, in the specific temporal and geographic context of the North American Lower Great Lakes region, empirically and quantitatively supporting the hypothesis that at least some unifacial stone tool spurs were intentionally created–and thus the class of artifacts we explicitly define as “unifacial stone tools” were indeed at times multifunctional implements–has important behavioral implications for Clovis foragers. Based on chronometric data and toolstone procurement and discard patterns, the Clovis occupation of this region is widely interpreted to represent a colonization pulse into a recently deglaciated area ([3]; [40]; [41]; [61]; [85]; [86]; [87];[103]; [104]; [105]; [106]; [107]; [108]; [109]). The property of multi-functionality would have increased the portability of the overall toolkit by reducing the number of artifacts needed to be carried, and thus allowing Clovis colonizing foragers to more quickly explore and learn the landscape to reduce uncertainty and risk in space and time ([72]; [110]; [111]). Furthermore, the property of multi-functionality would have allowed Clovis foragers to better respond to “situational contingencies” ([61]; [62]) as they arose during the possibly risky endeavor of colonizing a new and unfamiliar landscape ([112]).
